# Critical Involvement of TFIIB in Viral Pathogenesis

**DOI:** 10.3389/fmolb.2021.669044

**Published:** 2021-04-30

**Authors:** Michael J. O’Brien, Athar Ansari

**Affiliations:** Department of Biological Science, Wayne State University, Detroit, MI, United States

**Keywords:** virus, TFIIB, transcription, RNA polymerase II, pathogenesis, gene expression

## Abstract

Viral infections and the harm they cause to their host are a perpetual threat to living organisms. Pathogenesis and subsequent spread of infection requires replication of the viral genome and expression of structural and non-structural proteins of the virus. Generally, viruses use transcription and translation machinery of the host cell to achieve this objective. The viral genome encodes transcriptional regulators that alter the expression of viral and host genes by manipulating initiation and termination steps of transcription. The regulation of the initiation step is often through interactions of viral factors with gene specific factors as well as general transcription factors (GTFs). Among the GTFs, TFIIB (Transcription Factor IIB) is a frequent target during viral pathogenesis. TFIIB is utilized by a plethora of viruses including human immunodeficiency virus, herpes simplex virus, vaccinia virus, Thogoto virus, hepatitis virus, Epstein-Barr virus and gammaherpesviruses to alter gene expression. A number of viral transcriptional regulators exhibit a direct interaction with host TFIIB in order to accomplish expression of their genes and to repress host transcription. Some viruses have evolved proteins with a three-dimensional structure very similar to TFIIB, demonstrating the importance of TFIIB for viral persistence. Upon viral infection, host transcription is selectively altered with viral transcription benefitting. The nature of viral utilization of TFIIB for expression of its own genes, along with selective repression of host antiviral genes and downregulation of general host transcription, makes TFIIB a potential candidate for antiviral therapies.

## Introduction

Viruses have always been a threat to living creatures. Humans alone are the target of more than 200 viral species (Woolhouse et al., 2012; Knipe et al., 2013). The known viruses and the newly evolving strains, which are being discovered on a regular basis, have the potential to pose a global threat to humanity. They have caused pandemics in the past and the current COVID-19 pandemic is due to a recently evolved strain of coronavirus (Xie and Chen, 2020). It is necessary to identify, understand, and block replication of viruses to combat the hazards they pose. Viruses take advantage of their host to persist, replicate, and ultimately spread to a new host.

**FIGURE 1:**
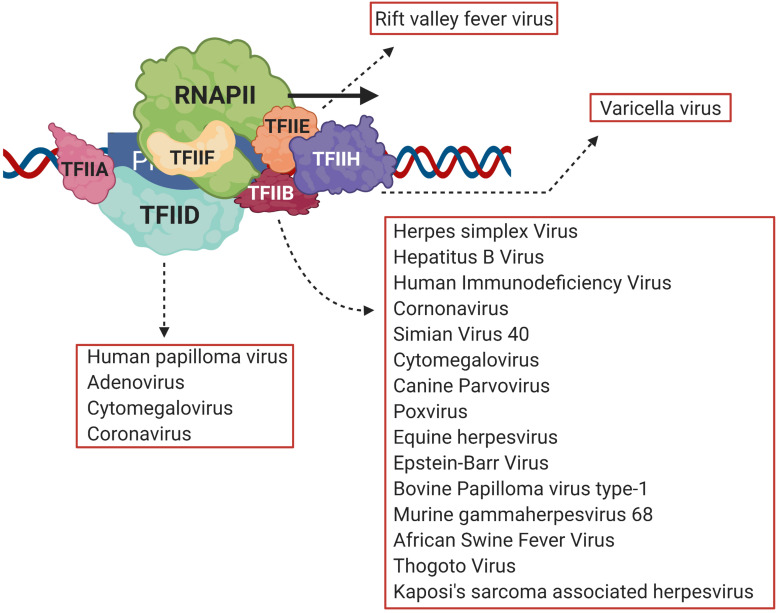
Viral transcriptional regulators target different general transcription factors, but TFIID and TFIIB are the prime targets. Viruses target general transcription factors such as TFIIB, TFIID, TFIIE and TFIIH in order to transcribe viral genes. TFIIB, however, is emerging as the most common target of many different viruses such as HIV, HSV, HCMV and HBV among others. Created with BioRender.com.

Necessary for viral infection is replication of the viral genome and production of viral proteins. Transcription of viral genes is the first step toward production of viral proteins (An et al., 2019; Liu et al., 2020). A number of DNA viruses and retroviruses use host transcription machinery to achieve this objective (Agostini et al., 1996; Gelev et al., 2014; Liu et al., 2020). Transcription is an essential biological process that results in production of RNA from the DNA template, a necessary step before the eventual production of proteins. At the center of transcription is the RNA polymerase (RNAP), the enzyme responsible for catalyzing RNA synthesis. In eukaryotes, RNA polymerase II (RNAPII) is responsible for synthesizing mRNAs, which subsequently are translated into proteins. The first step of transcription, known as initiation, involves recruitment of RNAPII by gene-specific transcription factors and a suite of general transcription factors (GTFs) on the promoter to form a preinitiation complex (PIC) (Krishnamurthy and Hampsey, 2009). The PIC consists of TFIID, TFIIA, TFIIB, TFIIF, TFIIE, and TFIIH together with RNAPII and Mediator complex (Woychik and Hampsey, 2002; Luse, 2014). Viruses target both gene-specific and GTFs to alter gene expression during pathogenesis. Among gene-specific factors IRF3, pro-inflammatory cytokines, NFκB and STATs are the most common viral targets (Lyles, 2000; Haas et al., 2018). A number of viruses also target GTFs to repress transcription of host anti-viral genes and to transcribe genes coding for viral proteins. The TATA-binding protein (TBP), which is a subunit of TFIID, is the target of HPV16 E7 protein, adenovirus E1A protein, and poliovirus 3C protein; TFIIE is the target of varicella virus IE63; while rift valley fever virus (RVFV) targets the TFIIH complex during viral pathogenesis (Figure 1; Maldonado et al., 2002; Dasgupta and Scovell, 2003; di Valentin et al., 2005; Kundu et al., 2005; Kalveram et al., 2011). TFIIB, however, is emerging as a critical viral target.

TFIIB is canonically involved in initiation of transcription by RNAPII and is an essential component of the PIC (Deng and Roberts, 2005; Luse, 2014). It is crucially important for recruitment of RNAPII on the promoter for initiation of transcription and is therefore taken advantage of by a number of viruses to transcribe their essential genes. Viral genomes encode transcriptional regulators, which alter viral and host transcription. A number of these viral regulatory proteins have been shown to interact with host TFIIB (Sundseth and Hansen, 1992; Smith et al., 1993; Yu et al., 1995; Agostini et al., 1996; Haviv et al., 1998; Jang et al., 2001; Vogt et al., 2008). The binding of TFIIB to a template is seemingly the critical, often rate limiting step in viral transcription where initiation will not occur if TFIIB is not present. The interaction of viral transcriptional regulators with TFIIB is therefore critical for transcription of protein-coding genes in a number of viruses. TFIIB has proven to be an important protein in viral transcription to such an extent that some viruses have evolved to possess proteins with structures similar to TFIIB in order to effectively transcribe their genes (Grimm et al., 2019; Cackett et al., 2020). Evidence suggests that TFIIB is not only intricately linked to viral pathogenesis, but is also selectively targeted by viruses to sustain viability. The widespread utilization of host TFIIB and TFIIB-like viral proteins by a number of viruses makes it an important, often essential, aspect of viral pathogenesis and a potential antiviral target.

## Viruses Target TFIIB to Enhance Expression of Their Genes and Repress Host Antiviral Genes

Viruses employ a two-pronged attack on host transcription machinery during pathogenesis. They exploit host transcription machinery to achieve expression of their own genes, and simultaneously downregulate transcription of host genes or gene products especially those linked to antiviral immune response (Gelev et al., 2014; Haas et al., 2018; Yang and You, 2020; Wang et al., 2020). A number of viruses use host transcription machinery to transcribe their protein-coding genes, and a number of them including herpes simplex virus-1 (HSV-1) target the general transcription factor TFIIB to achieve this goal. With RNAi knockdown of TFIIB, HeLa cells transfected with HSV-1 formed fewer plaques compared to the cells containing TFIIB (Gelev et al., 2014). Viral replication and plaque formation were reduced more than sevenfold upon knockdown of TFIIB. In the absence of TFIIB, transcription of most of the herpes genes examined was reduced, while expression of only a subset of host genes was adversely affected (Gelev et al., 2014). Likewise, human immunodeficiency virus (HIV), hepatitis B virus (HBV), human cytomegalovirus (HCMV), equine herpesvirus type 1 (EHV), and Epstein-Barr viruses (EBV) use TFIIB to enhance transcription of their genes (Figure 1; Caswell et al., 1993; Tong et al., 1995; Agostini et al., 1996; Haviv et al., 1998; Jang et al., 2001; Simmen et al., 2001).

Viral infection downregulates expression of a number of host genes related to anti-viral immune response. One such host gene normally induced upon viral infection is interferon regulatory factor 3 (IRF-3) (Lyles, 2000). IRF-3 along with IRF-7 are gene-specific transcription activators of type-I IFNs (IFN-α/β), which bind type-I IFN receptor present on the surface of the cell. Upon binding of IFN-α/β, the signal is transduced inside the cell by the signal transducer and activator of transcription STAT-1 and STAT-2. STATs translocate to the nucleus where they induce transcription of several hundred genes encoding proteins that protect the cell from viral infection. Viruses have evolved a variety of strategies to combat activity of such antiviral genes or gene products during pathogenesis. Polio and vesicular stomatitis viruses target TBP, while RVFV destabilizes the general transcription factor TFIIH to induce shut-off of host cell transcription (Yuan et al., 2001; Kundu et al., 2005; Kalveram et al., 2011). Gammaherpesviruses, including Kaposi’s sarcoma-associated herpesvirus (KSHV) and murine gammaherpesvirus 68 (MHV68), as well as orthomyxovirus Thogoto virus downregulate TFIIB to repress host gene expression (Hartenian et al., 2020). Gammaherpesvirus adversely affects general host transcription (Hartenian et al., 2020), while Thogoto virus selectively represses transcription of antiviral genes without affecting general host transcription. Thogoto virus specifically inhibits transcription of type-I IFNs (IFN-α/β), inflammatory cytokines, and antiviral effector genes (Jennings et al., 2005; Vogt et al., 2008; Haas et al., 2018). Thus, TFIIB is targeted in multiple ways during viral pathogenesis.

## Viral Expression Is Intertwined With TFIIB

An important issue is how viruses use TFIIB to alter transcription of their own genes and inhibit transcription of selected host genes. Evidence demonstrates that viruses accomplish this objective by physically interacting with TFIIB and recruiting it to the promoter of viral specific genes while preventing it from binding to selected host gene promoters. A number of viral transcriptional regulators like Vpr of HIV, HBx of Hepatitis B, VP16 and ICP4 of HSV, ML of Thogoto virus, IE2 of cytomegalovirus, E2TA of bovine papillomavirus type I, and IE of equine herpes virus exhibit a direct physical interaction with TFIIB (Table 1; Lin et al., 1991; Caswell et al., 1993; Rank and Lambert, 1995; Agostini et al., 1996; Haviv et al., 1998; Haas et al., 2018; Li et al., 2020). These viral transcription factors direct transcription of a subset of viral genes that are often vital to viability of the virus. Transcription of all viral genes, however, is not dependent on interaction of viral transcriptional factors with host TFIIB. The mechanism of regulation of transcription of viral and host genes by viral transcriptional regulators through their interaction with TFIIB is described in detail below.

**TABLE 1:** Viruses, their respective viral proteins, and the mechanism by which they regulate transcription.

**Virus**	**Viral protein**	**Mechanism of transcriptional regulation**
Herpes Simplex Virus I (HSV-I)	VP16	Direct interaction with TFIIB; TFIIB knockdown decreases HSVI gene transcription, viral replication, and plaque formation
Equine Herpesvirus 1 (EHV-1)	IE	Direct interaction with TFIIB; IE utilizes TFIIB to activate EHV-1 promoters
Thogoto Virus (THOV)	ML	Direction interaction with TFIIB; Downregulates TFIIB, represses antiviral gene transcription, and relocalizes TFIIB from nucleus to cytoplasm
Hepatitis B Virus (HBV)	HBx	Interacts with TFIIB to recruit RNAPII on viral promoters.
African Swine Fever Virus (ASFV)	ASFV-RNAP	Encodes a viral homolog similar to TFIIB
Human Immunodeficiency Virus Type 1 (HIV-1)	Vpr/Tat	Vpr: Direct interaction with TFIIB and induces transcriptionally active form of TFIIB. Tat: Forms ternary complex with TFIIB and human TAP protein facilitating interaction with transcriptional machinery
Epstein-Barr virus (EBV)	EBNA-2	Specific interaction with TFIIB crucial for transcription activation potential of EBNA-2
Murine gammaherpesvirus 68 (MHV68)	N/A	Reduced promoter occupancy of RNAPII due to viral induced degradation of RNAPII subunits and TFIIB
Kaposi’s sarcoma-associated herpesvirus (KSHV)	N/A	Reduced promoter occupancy of RNAPII due to viral induced degradation of RNAPII subunits and TFIIB
Poxviruses	vRNAP	Viral RNAP contains a subunit with homology to TFIIB.
Canine parvovirus (CBPV)	N/A	Sequesters TFIIB to viral induced nuclear compartment which is site of viral mRNA transcription
Simian virus 40 (SV40)	LSF	Increases rate of association of TFIIB to viral promoters leading to efficient PIC assembly
Cytomegalovirus	IE2	Direct interaction with TFIIB and likely crucial for transcriptional regulation of viral early promoters.
Coronavirus	N/A	TFIIB is a high confidence transcriptional target

Vpr and Tat: Vpr (Viral Protein R) is a highly conserved HIV-1 encoded protein required for replication, transcription and proliferation of the virus and is critical for viral pathogenicity. Vpr stimulates viral transcription, possibly due to its ability to interact with TFIIB (Agostini et al., 1996; Kino et al., 1999). *In vitro* studies have demonstrated a direct physical interaction of Vpr with TFIIB. Vpr binding brings about a change in the three-dimensional structure of TFIIB from “closed” to “open,” which is the transcriptionally active conformation (Agostini et al., 1996). Another HIV-1 protein Tat (Trans-Activator of Transcription), which drastically enhances viral transcription, also targets TFIIB to achieve its transactivation function (Yu et al., 1995). It forms a ternary complex with TFIIB and the human TAP protein. TAP protein binds strongly to a carboxy-terminal region of TFIIB as well as to the conserved activation domain of Tat. TAP acts as a bridge, facilitating interaction of the Tat transactivator with the host general transcription machinery through TFIIB (Yu et al., 1995). A direct protein-protein interaction has been observed between TFIIB and Tat (Veschambre et al., 1997).

VP16: Human herpes simplex virus 1 and 2 (HSV-1 and HSV-2) cause cold sores and genital herpes in humans. Both these viral species are common and contagious. Approximately 67% of the world population under the age of 50 has HSV-1 (James et al., 2020). Viral spread is dependent on the onset of the lytic cycle and reactivation. Viral tegument protein (VP16), which is the transcriptional activator of the immediate-early (IE) gene products (alpha genes), is the key activator of lytic infection (Mossman et al., 2000). VP16-induced transcription of IE genes acts as a regulatory switch; when it is on, it promotes lytic infection, and when it is off latent infection is favored. TFIIB is among several molecular targets of VP16 during transcription of IE genes. VP16 exhibits a physical interaction with both native and recombinant human TFIIB, but not with yeast or fly TFIIB (Lin et al., 1991; Gupta et al., 1996). A mutation in the VP16 activation domain, which adversely affects its transactivation function, reduced its binding to native TFIIB. Structural studies suggest that binding of VP16 brings about a conformational change in TFIIB that primes it for binding to the promoter-bound TBP leading to enhanced transcription of IE genes (Hayashi et al., 1998; Dion and Coulombe, 2003). The infected-cell polypeptide 4 (ICP4) protein of HSV acts as a transcriptional regulator, also affecting VP16 (Gu et al., 1995). The ICP4 protein has been observed to form a complex involving TFIIB in order to alter viral transcription (Smith et al., 1993; Gu et al., 1995).

HBx: Hepatitis B virus (HBV), which causes inflammation of the liver, also manipulates TFIIB during pathogenesis. HBV causes fatal liver infection and chronically infects more than 250 million people worldwide. The HBV minigenome persists in the nucleus of infected cells and is the template for production of four viral proteins. One of these proteins, HBx, is crucial for viral pathogenesis as it interacts with a number of host factors to facilitate viral replication and prevent antiviral response. One of the host proteins that HBx interacts with to enable viral transcription is TFIIB (Turton et al., 2020). HBx directly interacts with TFIIB through its B-finger motif (Haviv et al., 1998; Zhou et al., 2015). This interaction is critical for the coactivator function of HBx. TFIIB-HBx interaction facilitates recruitment of RNAPII on viral promoters leading to upregulation of viral RNA transcription.

IE1 and IE2: Human cytomegalovirus (HCMV) is another virus that targets TFIIB during pathogenesis. It is a common virus that infects people of all ages. Normally the infected people show only mild symptoms, but occasionally it causes serious disorders like mononucleosis and hepatitis. During viral pathogenesis, IE1 and IE2 proteins, which are the product of immediate early genes, transactivate a number of homologous (HCMV) and heterologous (non-HCMV) promoters. Transcription activation potential of IE2 is dependent on its interaction with two GTFs, TBP and TFIIB. IE2 exhibits a direct physical interaction with TFIIB (Caswell et al., 1993). The region of IE2 that mediates binding to TFIIB overlaps with that required for TBP binding. This is also the region linked to the transcriptional regulatory function of the protein. The IE2 gene produces three IE2 protein isoforms, IE2-86, IE2-60, IE2-40, late in infection. IE2-86 is essential for viral replication. An independent study demonstrated binding of IE2-86 to TFIIB under *in vitro* conditions (Xu and Ye, 2002). IE2-86-TFIIB interaction could be crucial for transcriptional regulation of viral early promoters, by facilitating downregulation of its own promoter and activating expression of many host cellular genes necessary for progression of viral infection.

ML: Thogoto virus is an orthomyxovirus that is transmitted to vertebrates through ticks. Thogoto virus targets TFIIB to repress transcription of host antiviral genes linked to innate immunity by a unique mechanism (Vogt et al., 2008; Haas et al., 2018). The ML (Matrix Long) protein of the virus physically interacts with TFIIB and relocalizes it to the cytoplasm (Haas et al., 2018). ML-mediated nuclear depletion of TFIIB represses transcription of genes that require *de novo* recruitment of RNAPII, while transcription of genes with paused polymerases continues unabated (Haas et al., 2018). Among host genes that require *de novo* recruitment of polymerases are antiviral immune response genes like pro-inflammatory cytokines, IRF3 and its regulated genes, as well as NFκB regulated genes. Sequestration of TFIIB by the ML protein therefore facilitates viral infection by selectively inhibiting host antiviral genes without affecting the bulk of general host transcription.

IE: In addition, equine herpesvirus type 1 (EHV-1) also targets TFIIB during pathogenesis (Jang et al., 2001). At least six viral transcriptional regulators including the IE (Immediate Early) protein regulates the coordinated expression of EHV-1 genes. The transcription activation potential of IE is dependent on its ability to interact with TFIIB. The IE interaction domain of TFIIB spans residues 125–174 in the first direct repeat of the protein. Transient transfection assays demonstrated that exogenous native TFIIB did not perturb transcription activation potential of the IE protein, but a TFIIB mutant that lacked the IE interactive domain adversely affected the ability of the IE protein to activate EHV-1 promoters (Albrecht et al., 2003). These results demonstrate that direct interaction of IE with TFIIB is essential for its ability to activate EHV-1 promoters.

## Viruses Target TFIIB Over TFIID and Other General Transcription Factors

Since transcription by RNAPII requires at least six GTFs, it is likely that GTFs other than TFIIB are also targeted during viral pathogenesis. There are reports of viruses interacting with the TFIID subunit TBP, TFIIE and TFIIH to achieve transcription of viral genes and turn off host transcription. TBP and TFIIB, however, have emerged as the preferred target of viral transcriptional regulators (Sundseth and Hansen, 1992; Tong et al., 1995; Ihalainen et al., 2012). Transcription regulators of some viruses like SV40, Epstein-Barr virus and canine parvovirus (CPV) interact with both TFIIB and TBP. TFIIB, however, is more critical for viral pathogenesis. In the case of gammaherpesvirus, despite both TFIIB and TFIIA being the viral targets, TFIIB is the key factor for viral transcription (Hartenian et al., 2020).

LSF (Late Simian virus 40 transcription Factor) is a cellular transcriptional activator of SV40 that dramatically increases transcription from viral major late promoters. LSF enhances transcription by facilitating assembly of the PIC on major late promoters. LSF, however, does not affect binding of TFIID to the promoter. Instead, it increases the rate of association of TFIIB, which leads to efficient PIC assembly and increased transcription of SV40 genes (Sundseth and Hansen, 1992). Similarly, Epstein-Barr virus nuclear antigen 2 (EBNA-2) has an acidic domain, which is essential for the transcription activation potential of EBNA-2 (Tong et al., 1995). EBNA-2 exhibits specific interaction with TFIIB, while it’s binding affinity for TBP is much less (Tong et al., 1995). The EBNA-2-TFIIB interaction is crucial for transcription activation potential of EBNA-2 during the viral life cycle.

Canine parvovirus (CPV) infection leads to formation of a proteinaceous sub-compartment within the nucleus, which is the site of transcription of viral mRNA. Fluorescence recovery after photobleaching (FRAP) revealed accumulation of both TBP and TFIIB in the nuclear sub-compartment during viral infection. TBP and TFIIB, however, exhibited different kinetics of diffusion and binding affinities to the nuclear sub-compartment (Ihalainen et al., 2012). The binding affinity of TBP to the nuclear body area decreased upon viral infection while that of TFIIB slightly increased. The measured binding time of TFIIB reflected the time scale of TFIIB association with the PIC and recruitment of RNAPII to the promoter, indicating selective usefulness of TFIIB over TBP for transcription of CPV genes in the nuclear body area.

Kaposi’s sarcoma-associated herpesvirus (KSHV) and murine gammaherpesvirus 68 (MHV68) infection leads to mRNA-decay induced repression of transcription of antiviral genes and overall general transcription of host cells. Viral induced downregulation of host transcription was due to the genomewide decrease in RNAPII promoter occupancy (Hartenian et al., 2020). Reduced promoter occupancy was attributed to viral induced degradation of subunits of RNAPII and TFIIB. The amount of TFIIA also registered a slight decline upon viral infection. There was, however, absolutely no decrease in the level of TBP in the cell. Clearly, SV40, Epstein-Barr virus, canine parvovirus and gammaherpesviruses target TFIIB over TBP or TFIIA during viral pathogenesis.

## Viruses Have Evolved Proteins Similar to TFIIB

Some viruses have evolved proteins with a structure and function similar to that of TFIIB, resulting in less reliance upon the host. Vaccinia virus, which is a prototype poxvirus, encodes its own RNAP (vRNAP). The vRNAP is a multi-subunit protein capable of carrying out transcription in the cytoplasm, not reliant upon host RNAPII to transcribe viral genes (Moss, 2013; Grimm et al., 2019). Replication in the cytoplasm not only rules out the utilization of host RNAPII, but of natively localized host TFIIB as well. The vRNAP complex consists of eight subunits with varying degrees of homology to RNAPII and one subunit, Rap94, displays homology to TFIIB (Grimm et al., 2019; Hillen et al., 2019; Liu et al., 2020). A central region of Rap94 possesses a “B-homology region” containing elements homologous to eukaryotic TFIIB B-ribbon, B-cyclin, and B-reader domains. These regions allow Rap94 to interact with RNAPII, bind to DNA, and determine start site selection in a manner similar to host TFIIB (Nikolov and Burley, 1997; Bushnell et al., 2004; Weinzierl and Wiesler, 2011; Sainsbury et al., 2013; Grimm et al., 2019; Hillen et al., 2019). These similarities suggest that Rap94 likely functions in transcription initiation, bypassing the need to utilize host TFIIB (Grimm et al., 2019). African Swine Fever Virus (ASFV) also encodes a vRNAP with subunits exhibiting homology to RNAPII subunits and TBP. One of the subunits also exhibits remarkable structural and functional similarities to TFIIB (Cackett et al., 2020). For such viruses like vaccinia virus and ASFV, which assemble a viral PIC using a vRNAP complex, the critical usage of host TFIIB is bypassed, as they have evolved TFIIB-like proteins. Concerning the gammaherpesvirus, host TFIIB is used in conjunction with the TFIIB-like viral protein for transcribing viral genes. Host TFIIB is required for early transcription of viral genes that produces components of the vPIC. Once vPIC proteins are synthesized, the requirement of host TFIIB for transcription of late viral genes is bypassed (Nandakumar and Glaunsinger, 2019). Thus, early transcription during viral life cycle is vPIC-independent, while late transcription is vPIC-dependent. The presence of these TFIIB-like proteins in viral transcription complexes simply reflects the vital role of TFIIB in viral gene expression.

## Discussion

TFIIB is a general transcription factor that is essential for initiation of transcription from a majority of RNAPII-transcribed genes in eukaryotes. Viruses can target any general transcription factor to inhibit transcription of antiviral genes for successful pathogenesis. The general target of most viruses, however, are TFIID and TFIIB. The preferential targeting of these GTFs prevents assembly of PICs at an early stage prior to the recruitment of RNAPII. Recent studies have uncovered rather unexpected novel roles of TFIIB in the transcription cycle. The studies have revealed that TFIIB is not merely an initiation factor but plays pleiotropic roles in the transcription cycle (Wang et al., 2010; Medler et al., 2011; Tan-Wong et al., 2012). TFIIB affects gene architecture by facilitating interaction of the terminator with the promoter of the cognate gene during transcription (Medler et al., 2011). The promoter-terminator interaction results in the formation of a looped gene architecture (Ansari and Hampsey, 2005). Gene looping affects termination, reinitiation and promoter directionality (Grzechnik et al., 2014; Al-Husini et al., 2020). Thus, viruses have the potential to affect multiple aspects of the transcription cycle by targeting TFIIB. This could be one of the reasons why viral transcriptional regulators prefer TFIIB over TBP and more so than other GTFs during viral pathogenesis. Future research must focus on which of the TFIIB-dependent processes described above are the target of viral transcriptional regulators during pathogenesis.

The importance of TFIIB in completion of the viral life cycle is corroborated by the fact that multiple viruses have evolved proteins with structural and functional similarity to host TFIIB. Bypassing the need for host TFIIB, these viruses are now self-sufficient in terms of their TFIIB requirement, thus demonstrating the critical role of TFIIB in viral transcription. It is not surprising that TFIIB was also identified along with a number of other proteins as a high confidence transcriptional target (HCT) during infection by the current coronavirus (Ochsner et al., 2020). Taken together, selective downregulation of viral transcription without compromising host gene expression, specific viral targeting of TFIIB over other GTFs, and evolution of viral TFIIB-like proteins are compelling evidence for the critical role of TFIIB in viral pathogenesis. Involvement of TFIIB in pathogenicity of multiple human viruses by altering viral and host gene expression makes TFIIB a potential target of antiviral therapies. Future research concerning the extent of abrogation or inhibition of TFIIB necessary to invoke negative viral responses while maintaining normal host function would further foretell how TFIIB may be targeted to control viral infection. This is likely to be accomplishable as organisms or cells with mutant or inhibited TFIIB have been shown to be viable for study (Gelev et al., 2014). A three-dimensional structure of viral transcription regulators with TFIIB will elucidate the region of TFIIB essential for viral pathogenesis. The regions of TFIIB targeted by virus but not critical for host transcription may be the ideal drug target for future antiviral therapies. The viral TFIIB-like proteins of gammaherpesvirus and ASFV are also the potential drug targets for stopping the lifecycle of these viruses as selective inhibition of vPIC-dependent transcription may stop viral replication without adversely affecting host cell functions.

## Author Contributions

AA conceptualized and edited the manuscript. MJO wrote the original draft. Both authors contributed to the article and approved the submitted version.

## Conflict of Interest

The authors declare that the research was conducted in the absence of any commercial or financial relationships that could be construed as a potential conflict of interest.
